# Parent Experiences of a Pilot Functional Neurological Disorders Clinic at Great Ormond Street Hospital (GOSH)

**DOI:** 10.1192/bjo.2024.469

**Published:** 2024-08-01

**Authors:** Talia Eilon, Sacha Evans, Suresh Pujar, Lily Orme, Abbie Smith

**Affiliations:** 1 Great Ormond Street Hospital, London, United Kingdom; 2 University of Durham, Durham, United Kingdom

## Abstract

**Aims:**

There are a shortage of specialist services available for Functional Neurological Disorders, especially within the paediatric population. Patients and families often find themselves falling within the borderland between medical and psychiatric services. Functional symptoms can cause significant morbidity and disruption to the lives of children and young people, impacting their access to education and social lives. Early diagnosis and explanation of FND is a mainstay of treatment, and is associated with positive outcomes. A Functional Neurological Disorder pilot MDT clinic was set up within Great Ormond Street Hospital, with the aim to provide a one-off therapeutic assessment and psychoeducation. We surveyed families who attended the clinic to assess their experiences and outcomes.

**Methods:**

A pilot clinic was set up for patients referred within GOSH with a confirmed diagnosis of FND. The Multidisciplinary team consisted of a CAMHS psychiatrist, paediatric neurologist, physiotherapist and occupational therapist. Patients received a one-off outpatient consultation to discuss FND symptoms and background history. Clinicians provided psychoeducation for patients and families about the diagnosis and devised treatment plans including follow-up assessments, onward referral to local services and a consultation with teams where appropriate. A follow-up survey was conducted using semi-structured telephone interviews and patient satisfaction questionnaires. Questionnaires were scored using a Likert rating scale (1: very dissatisfied – 5: very satisfied). Parents were asked about their understanding of the FND diagnosis and about their experiences of support from local teams.

**Results:**

25 patients diagnosed with FND were referred to the clinic. Of those, 20 patients took up the consultation. Patients presented with range of functional syndromes. 15 families consented to follow-up interviews. Parents rated their experience at the FND clinic highly (median score 5 – very satisfied). They were very dissatisfied with follow up care (median score 1). Only one patient remained under CAMHS at the time of follow up. 3 families had sought support privately. Parents subjectively rated their children's symptoms at follow up as: much worse (3); a bit worse (1); the same (7); a bit better (2) and much better (2).

**Conclusion:**

Patients and parents demonstrated high levels of satisfaction with the one-off therapeutic assessment. The majority of parents reported that the GOSH consultation helped them to understand the diagnosis of FND. All families felt they had received inadequate support from primary care, local CAMHS services and schools. Patients who struggled to access support from CAMHS/ school were less likely to experience any improvement in FND symptoms and had poorer levels of functioning.

